# The tiny giants of regeneration: MSC-derived extracellular vesicles as next-generation therapeutics

**DOI:** 10.3389/fcell.2025.1612589

**Published:** 2025-07-17

**Authors:** Tianhe Zhang, Liang Zhang, Xiaoxue Ma, Wenliang Song

**Affiliations:** ^1^Department of Pediatrics, Shengjing Hospital of China Medical University, Shenyang, China; ^2^Department of Neonatology, The Frist Hospital of China Medical University, Shenyang, China; ^3^Department of Pediatrics, The Frist Hospital of China Medical University, Shenyang, China

**Keywords:** extracellular vesicles, mesenchymal stem cells, exosomes, therapy, bioengineering, animals, humans, clinical trial

## Abstract

Mesenchymal stem cell-derived extracellular vesicles (MSC-EVs) are revolutionizing the field of regenerative medicine, becoming the core carriers of next-generation acellular therapeutic strategies. In contrast to traditional mesenchymal stem cell therapy, these nanoscale “regenerative tiny giants” offer significant advantages, including low immunogenicity, efficient biological barrier penetration, and stable storage. As natural bioactive molecular carriers, MSC-EVs precisely regulate the inflammatory response, angiogenesis, and tissue repair processes in target tissues by delivering functional RNA, proteins, and other signaling elements. They have demonstrated multidimensional therapeutic potential in diseases such as bone and joint regeneration, nerve function reconstruction, myocardial repair, and skin wound healing. Worldwide, 64 registered clinical trials have preliminarily validated the safety and applicability of MSC-EVs across various diseases. Notably, they have shown significant progress in treating severe coronavirus disease 2019 (COVID-19), ischemic stroke, and complex wound healing. However, the lack of standardization in production processes, insufficient targeting for *in vivo* delivery, and the scarcity of long-term biodistribution data remain core bottlenecks limiting the clinical translation of MSC-EVs. Future interdisciplinary technologies, including 3-dimensional (3D) dynamic culture, genetic engineering, and intelligent slow-release systems, are expected to facilitate the transition of MSC-EVs from the lab to large-scale applications. This shift may transform “injectable regenerative factors” into “programmable nanomedicines”, offering new solutions for precision medicine.

## 1 Introduction

In recent decades, stem cell therapy has gained considerable interest for its potential to treat various diseases and injuries. Among the various types of stem cells, mesenchymal stem cells/mesenchymal stromal cells (MSCs) are the most researched, especially for their roles in regenerative medicine and tissue engineering (Gowen et al., 2020). Mesenchymal stromal cells are generally defined as spindle-shaped cells with plastic adhesive properties (Horwitz et al., 2005). Mesenchymal stem cells are a subpopulation of MSCs that have demonstrated stem cell activity based on rigorous criteria (Keating, 2006). In 1970, Alexander Friedenstein first identified MSCs as clonal progenitor cells capable of differentiating into fibroblasts and other mesodermal cell types (Friedenstein et al., 1970). MSCs can be isolated from various sources, including fetal tissues (umbilical cord and placenta) and adult tissues (bone marrow, adipose tissue, liver, skin, synovium, and dental pulp) (Fan et al., 2020). It is hypothesized that MSCs will migrate to the injury site after administration and transplantation, where they will regulate inflammatory responses and differentiate into functional cells to repair damaged tissue (Trigo et al., 2024). Many preclinical and clinical studies have evaluated the effectiveness of MSCs in tissue repair and regeneration (Kolenc and Maličev, 2024).

As of now, more than 2,300 human clinical trials involving MSCs have been registered, focusing on conditions like osteoarthritis, traumatic brain injury, septic shock, diabetic nephropathy, respiratory infections and tumors. https://www.clinicaltrials.gov/(search term: “mesenchymal (stem or stromal) cell”). However, moving MSC therapies from preclinical studies to clinical use has faced many challenges. Although preclinical findings in various animal models have shown promise, most registered clinical trials have not achieved the expected objectives (Zhou et al., 2021). The effectiveness of MSCs treatment depends on several factors, such as how well the cells home to the injury site, adhere, survive, retain, modulate the immune response, promote angiogenesis, implant, and integrate (Chang et al., 2021). Consequently, poor and inconsistent quality control in areas like immunocompatibility, stability, heterogeneity, differentiation, and migration capacity, along with concerns about infusion toxicity and tumorigenicity, have led to setbacks in clinical development (Zhou et al., 2021; Prockop et al., 2010). This indicates that significant challenges in preclinical and clinical applications remain to be addressed.

Over the past decade, there has been a significant shift in understanding that MSCs primarily deliver therapeutic effects by releasing paracrine factors, instead of migrating and differentiating in injured tissues (Johnson et al., 2021; Bazzoni et al., 2020). The secretome of these cells encompasses soluble factors such as growth factors, cytokines, chemokines, and hormones, in addition to insoluble factors contained within extracellular vesicles (EVs), which are one of the main sub-secretory effectors (Miclau et al., 2023). Theoretically, EVs are capable of executing functions akin to those of their parent cells and may serve as valuable substitutes for these cells, thereby presenting several advantages over MSCs (Soler-Botija et al., 2022).

The distinct benefits of EVs compared to conventional stem cell therapies include (Kou et al., 2022; Draguet et al., 2023): i. Enhanced safety, as EVs do not replicate once administered in the body, which significantly mitigates the risk of carcinogenesis. As nanoparticles, EVs are biocompatible and have low immunogenicity. This allows them to cross protective barriers, such as the blood-brain barrier, without causing embolism or transmitting infections; ii. Ease of storage: EVs can be preserved at −80°C for extended periods without losing biological activity, even after multiple freeze-thaw cycles; iii. Versatile administration routes, including topical application, intravenous injection, and oral delivery, alongside their capacity for encapsulation, thereby functioning as an effective drug delivery system; iv. Greater cost-efficiency, as products derived from secretory vesicles can be continuously produced by immortalized cell lines, enabling the acquisition of sufficient quantities and reducing the associated time and costs of expanding and maintaining cloned cell lines. These advantages make EVs exceptionally promising for clinical applications. As of January 2025, there are 64 registered clinical trials of mesenchymal stem cell-derived extracellular vesicles (MSC-EVs) for various diseases, which can be found on ClinicalTrials.gov (search term: “mesenchymal stem cell-extracellular vesicles/exosomes”), as shown in Table 1.

**TABLE 1:** Clinical trials of mesenchymal stem cell-derived extracellular vesicles therapy.

NCT number	Conditions	Phases	Enrollment	Study status	Locations
NCT05261360	Knee Injury	2	30	Recruiting	Turkey
NCT04223622	Osteoarthritis	NA	36	Completed	Italy
NCT06607900	Neurodegenerative Diseases	1	100	Not yet recruiting	China
NCT06598202	Amyotrophic Lateral Sclerosis	1/2	38	Recruiting	China
NCT05669144	Myocardial Infarction	1/2	20	Unknown	Iran
NCT05354141	Acute Respiratory Distress Syndrome	3	970	Recruiting	United States
NCT05787288	COVID-19 Pneumonia	1	240	Recruiting	China
NCT05808400	Long COVID-19 Syndrome	1	80	Recruiting	China
NCT05871463	Decompensated Liver Cirrhosis	2	15	Recruiting	Iran
NCT05402748	Fistula Perianal	1/2	80	Unknown	Iran
NCT05130983	Crohn Disease	1	10	Active not recruiting	United States
NCT05176366	Ulcerative Colitis	1	10	Active not recruiting	United States
NCT05813379	Skin Rejuvenation	1/2	20	Recruiting	Iran
NCT04173650	Dystrophic Epidermolysis Bullosa	1/2	10	Recruiting	United States
NCT06482541	Androgenetic Alopecia	1	100	Not yet recruiting	United States
NCT06221787	Melasma	NA	80	Recruiting	China
NCT02138331	Diabetes Mellitus Type 1	2/3	20	Unknown	Egypt
NCT06812637	Diabetic Foot Ulcer	1	110	Completed	Egypt
NCT06072794	Premature Ovarian Insufficiency	1	9	Suspended	United States
NCT03562715	Preeclampsia	NA	200	Completed	NA
NCT04213248	Dry Eye Disease	1/2	27	Unknown	China
NCT03437759	Macular Holes	1	44	Unknown	China
NCT05413148	Retinitis Pigmentosa	2/3	135	Unknown	Turkey
NCT06764004	Apical Periodontitis	1/2	45	Not yet recruiting	Turkey
NCT06245746	Acute Myeloid Leukemia	1	9	Not yet recruiting	NA
NCT06536712	Rectal Cancer	1	20	Not yet recruiting	Iran
NCT04544215	Drug-resistant	1/2	60	Suspended	China
NCT04356300	Multiple Organ Failure	NA	60	Not yet recruiting	NA

Data obtained from ClinicalTrials.gov using ‘mesenchymal stem cell-extracellular vesicles’ and ‘mesenchymal stem cell-extracellular exosome’ as keywords, as of 2025–01-01. The categorization of diseases follows the system by ClinicalTrials.gov.

NA, not applicable.

This review categorizes MSC-EVs based on different human systems. It comprehensively elaborates on their mechanisms of action and the latest research progress in various diseases, focusing on their regulatory effects on the immune system and tissue repair. Subsequently, the review analyzes the current status of clinical trials and discusses challenges in large-scale production and targeted drug therapy. It then proposes feasible improvement methods to provide new ideas for future clinical translation.

## 2 Overview of MSC-EVs

### 2.1 EVs biogenesis

EVs are vesicles with a double-layer lipid membrane produced by cells. They facilitate intercellular communication and play significant roles in both physiological and pathological contexts. EVs can be classified into three main types based on their biological origin (Bazzoni et al., 2020; Soler-Botija et al., 2022; Tsiapalis and O'Driscoll, 2020): i. Exosomes, which are small vesicles (30–150 nm) formed from endocytosing cells; ii. Microvesicles (100 nm–1 μm), which bud directly from the plasma membrane; and iii. Apoptotic bodies (100 nm–5 μm), which are created through membrane blebbing during apoptosis. The cargo carried by EVs includes DNA, both coding and non-coding RNA (such as mRNA, miRNA, lncRNA, and circRNA), as well as a variety of soluble bioactive molecules, all of which mediate distinct functions based on their cellular origins (Al Halawani et al., 2022).

**FIGURE 1:**
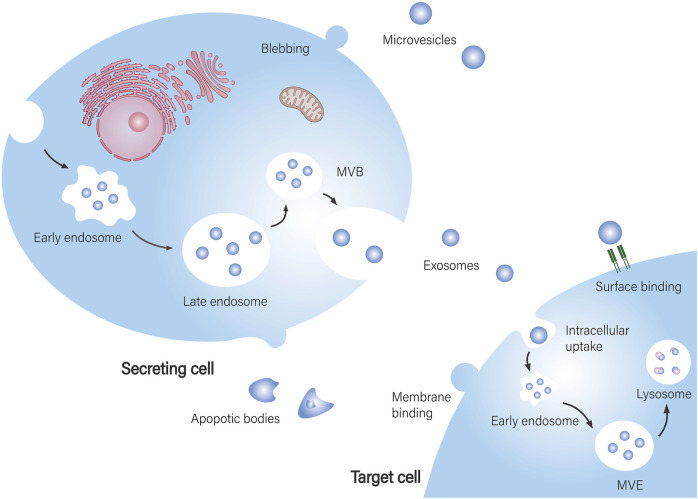
Biogenesis of EVs and their interaction with target cells. EV biogenesis and secretion: Exosomes are assembled in multivesicular bodies (MVB), where specific cargo is sorted into exosomes and subsequently released into the extracellular space. Microvesicles are formed by budding from the cell membrane. Apoptotic bodies are produced by apoptotic cells. EVs are taken up by recipient cells: EVs fully fuse with the recipient cell membrane, releasing their contents directly into the cytoplasm of the target cell. After internalization by the target cell, they reach multivesicular endosomes (MVE) and are further degraded and recycled through lysosomes. EVs bind to signaling receptors on target cells to regulate signal transduction.

The biogenesis of EVs includes four stages: initiation, endocytosis, formation of multivesicular bodies, and release (Fernández-Francos et al., 2021). Interactions between EVs and target cells can be divided into three main types (Miclau et al., 2023): i. Complete fusion with the target cell membrane, allowing direct release of contents into the cytoplasm, which is the primary way EVs exert their effects; ii. Internalization, where EVs enter lysosomes and release their cargo, influencing cellular activities; iii. Binding of transmembrane proteins on EVs to receptors on target cells, which affects signal transduction and cellular function (Figure 1).

### 2.2 MSC-EVs bioengineering applications

**FIGURE 2:**
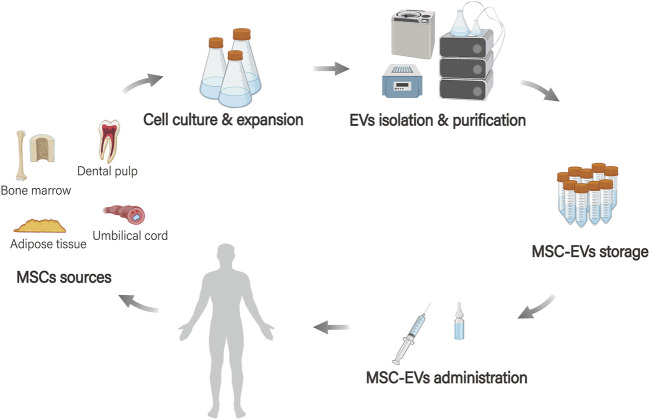
MSC-EVs bioengineering applications. MSCs originate from various tissue sources (such as adipose tissue, bone marrow, dental pulp, and umbilical cord tissue), and EVs are extracted after processes such as cell culture, amplification, and separation purification. These vesicles are then stored and the MSC-EVs serve as a drug delivery system in the body.

The production of MSC-EVs involves several steps: first, cells are extracted from different sources. Next, these cells undergo culture and expansion, followed by separation, purification, and storage (Maumus et al., 2020; Ma et al., 2024; Nagelkerke et al., 2021). After the production process, MSC-EVs are utilized as a drug delivery system in the body, which includes the stages of drug loading, delivery, and targeted therapy (Rezaie et al., 2022) (Figure 2).

The source of MSCs and culture conditions significantly impact vesicle production. Variations in donor characteristics, including health status, genetics, gender, and age, can lead to significant heterogeneity (Zhou et al., 2021). Furthermore, the proliferation and differentiation potential of cells sourced from diverse tissues, including bone marrow, adipose tissue, and dental pulp, exhibit notable differences (Costela-Ruiz et al., 2022). Additionally, the culture environment, including oxygen tension, substrate types, extracellular signals, inflammatory stimuli, and culture media composition, along with genetic or exosomal modifications, can enhance the survival, targeting, and therapeutic efficacy of these cells (Costa et al., 2021).

The isolation and purification of MSC-EVs is a critical step to ensure their quality and functionality. Although there is no standardized method for separation and purification, ultracentrifugation remains the most commonly used technique (Zarovni et al., 2015). While prevalent in laboratory settings, this method is often criticized for its labor intensity and the large sample volumes it requires (Zaborowski et al., 2015). Alternative strategies, including filtration, ultrafiltration, size-exclusion chromatography, immunoprecipitation, and precipitation using reagents like polyethylene glycol, have been explored with varying outcomes in terms of purity and yield (Doyle and Wang, 2019). Following isolation and purification, these vesicles are usually preserved at −80°C in a phosphate-buffered saline solution (Pincela Lins et al., 2023).

MSC-EVs have become ideal carriers for drug delivery systems due to their biocompatibility, low immunogenicity, ability to cross biological barriers (such as the blood-brain barrier), and their capacity for drug loading or gene editing loading, as well as being targeted and taken up by cells (Rezaie et al., 2022). Currently, significant efforts focus on designing drug carriers that can deliver treatments directly to target sites while reducing dosages and side effects. These carriers are intended for use in chemotherapy or anti-inflammatory therapies, and their effectiveness has been confirmed in various studies (Zhang et al., 2022; You et al., 2021). Notably, drugs like paclitaxel and doxorubicin have been successfully encapsulated and delivered to specific tissues in various tumor models, reducing side effects and improving treatment results (Agosti et al., 2024).

## 3 Therapeutic potential of MSC-EVs

### 3.1 Musculoskeletal diseases

Musculoskeletal disorders (MSDs) affect bones, joints, muscles, and soft tissues. They are typically caused by injury or degeneration and can severely limit a patient’s mobility (Abreu et al., 2021). The prevalence of MSDs is increasing due to an aging population. These disorders are the leading cause of chronic diseases that result in disability and require rehabilitation worldwide (Cieza et al., 2021). MSDs primarily include fractures, osteoporosis, osteoarthritis, rheumatoid arthritis, intervertebral disc degeneration, tendon injuries, ligament injuries, and related conditions. These injuries are accompanied by a lot of pain and usually have no clear cure, while existing treatments are often used to relieve pain (Malekpour et al., 2022). MSC-EVs help maintain bone metabolic balance and promote the remodeling and mineralization of the extracellular matrix (ECM) in musculoskeletal tissues (Herrmann et al., 2020). They also regulate immune responses, stimulate angiogenesis, and support bone innervation, which contributes to the homeostasis and regeneration of these tissues (Wang et al., 2024a). MSC-EVs are gaining attention as a new therapeutic strategy in the treatment of orthopedic diseases.

In the treatment of fractures, MSC-EVs facilitate bone healing by adjusting the inflammatory environment, encouraging osteogenic progenitor cells to differentiate into osteoblasts, promoting new blood vessel formation, and improving the migration of circulating mesenchymal stem cells to the injury site (Malekpour et al., 2022). In a mouse fracture model, exosomes delivered via hydrogel microparticles effectively maintain their activity and half-life. This method is also linked to the overexpression of miR-29a, which speeds up fracture healing by linking bone formation and blood vessel growth (Pan et al., 2024). In another *in vitro* experiment, exosomes from adipose-derived stem cells under hypoxia, through miR-21-5p, stimulate the proliferation, migration, and angiogenic potential of human umbilical vein endothelial cells to treat osteoporotic fractures (Li X. et al., 2024).

In the treatment of osteoporosis, MSC-EVs help restore bone formation mediated by osteoblasts and maintain bone homeostasis regulated by osteoclasts, while also reducing bone loss (Jia et al., 2023). In an osteoporotic mouse model, miR-22-3p delivered by bone marrow mesenchymal stem cell-derived EVs (BMSC-EVs) was found to potentially lead to inhibition of the MYC/PI3K/AKT pathway, thereby promoting osteogenic differentiation through FTO inhibition (Zhang et al., 2020). In the ovarian removal mouse osteoporosis model, exosomes loaded with AMG487 increased macrophage uptake by blocking osteoclast recruitment, reducing osteoclast differentiation, and hindering macrophage migration *in vitro*, effectively preventing bone loss (Kang et al., 2024).

Osteoarthritis involves the destruction of articular cartilage, sclerosis of subchondral bone, abnormal bone remodeling, and inflammation of the synovial tissue (Yao et al., 2023). MSC-EVs promote chondrocyte proliferation and migration, inhibit chondrocyte apoptosis and senescence, enhance matrix synthesis, prevent cartilage destruction, and influence immune regulatory signaling (You et al., 2023; Nguyen et al., 2021; Boulestreau et al., 2021). Liu et al. demonstrated that EVs from human umbilical cord mesenchymal stem cells can inhibit cell death via the miR-223/NLRP3/apoptosis pathway, enhance chondrocyte metabolism *in vitro*, and protect osteoarthritis rat articular cartilage from damage (Liu W. et al., 2023). Ye et al. found that miR-3960 downregulates PHLDA2, which reduces the inflammatory response in chondrocytes and decreases ECM degradation (Ye et al., 2022). Additionally, Li et al. reported that injecting mitochondria-rich extracellular vesicles from cultured human synovial fluid-derived mesenchymal stem cells into the knee joints of osteoarthritis rats downregulated oxidative stress markers and senescence-associated proteins. This treatment inhibited chondrocyte senescence and effectively alleviated articular cartilage degeneration (Li X. et al., 2025).

In tendon and ligament repair, MSC-EVs regulate the immune system primarily through macrophage polarization, promote angiogenesis, enhance the proliferation and migration of fibroblasts, and facilitate the synthesis of the extracellular matrix (Wu et al., 2024). For instance, in a rat model of Achilles tendon injury, miR-29a-3p improved the tendon’s pathological structure, enhanced the specific matrix components, and optimized its biomechanical properties via the PTEN/mTOR/TGF-β1 signaling pathway (Yao et al., 2021). In a rat model of anterior cruciate ligament (ACL) reconstruction, bone marrow mesenchymal stromal cell-derived exosomes (BMSC-Exos) promote the polarization of M1 to M2 macrophages via miR-23a-3p, reduce the early inflammatory response at the tendon-bone interface, and enhance early healing after ACL reconstruction (Li Z. et al., 2022).

### 3.2 Neurological diseases

Neurological diseases are the primary cause of global disability, leading to various medical and social issues, along with a substantial economic burden due to their complex and expensive treatments (Liu Y. Y. et al., 2023). Neurological diseases can be categorized into three main types: neurodegenerative diseases, such as Alzheimer’s disease (AD), Parkinson’s disease, and multiple sclerosis; traumatic diseases, including traumatic brain injury (TBI), spinal cord injury, and peripheral nerve injury; and vascular diseases, like stroke. MSC-EVs can alleviate glial cell proliferation, decrease neuronal death, modulate pro-inflammatory signaling, and lessen cognitive, behavioral, and motor impairments (Reed and Escayg, 2021). Furthermore, EVs are particularly beneficial in treating neurological diseases because they can cross the blood-brain barrier and effectively deliver their contents to receptor cells (Yuan et al., 2022).

AD is marked by the buildup of amyloid-β (Aβ) in extracellular plaques and hyperphosphorylated tau in intracellular tangles (Ising and Heneka, 2018). MSC-EVs can enhance both pathological changes and cognitive abilities in AD mice. They achieve this by clearing abnormal protein accumulation, repairing damaged synapses, inhibiting neuronal apoptosis, regulating microglial polarization, and reducing oxidative stress (Zhang W. et al., 2024; Ye et al., 2023). For example, MSC-EVs containing miR-206-3p antibodies increased brain-derived neurotrophic factor levels in AD mice after nasal administration. This improved learning and memory deficits, promoted neurogenesis and synaptic plasticity in the hippocampus, and reduced Aβ deposition (Peng et al., 2024). In the AD rat model, BMSC-Exos have shown the potential to enhance memory, reduce Aβ accumulation, promote neurogenesis, and alleviate astrocyte inflammation by regulating the PI3K/Akt/mTOR pathway, autophagy, and neuroinflammation (Ebrahim et al., 2024).

TBI results in immediate neuronal and blood-brain barrier damage, followed by glial proliferation and injury that begin within minutes (Maas et al., 2022). Acute injury and neurovascular damage can lead to myelin cell aggregation and T cell recruitment, potentially resulting in chronic neurodegeneration (Jarrahi et al., 2020). MSC-EVs promote nerve and vascular regeneration by reducing neuronal cell death and inflammatory responses, facilitating axonal and myelin reformation, enhancing synaptic plasticity, alleviating TBI, and promoting neural repair (Zhou et al., 2023; Xiong et al., 2024). For example, exosomes derived from human umbilical cord mesenchymal stem cells (HUCMSC-Exos) reduce neuronal cell death, inhibit inflammation, suppress ferroptosis, and play a key role in neuroprotection through the lncRNA TUBB6/Nrf2 pathway following TBI (Zhang L. et al., 2024). Additionally, in a TBI mouse model, high doses of BMSC-EVs were administered intranasally. This treatment reduced the release of pro-inflammatory cytokines by inhibiting chronic activation of the NLRP3-p38/MAPK signaling pathway. It also prevented long-term cognitive and emotional disorders and improved brain dysfunction caused by chronic inflammation (Kodali et al., 2023).

Stroke can cause nerve cell death and release factors like damage associated molecular patterns (DAMPs), which trigger local inflammation in the affected brain area. This inflammation worsens blood-brain barrier damage, microvascular failure, brain edema, oxidative stress, and directly induces further nerve cell death, thereby exacerbating secondary brain injury (Shi et al., 2019). MSC-EVs can reduce neuronal damage, improve glial cell function, and decrease the size of cerebral infarction in stroke models by promoting cell recycling processes and regulating inflammation (Li Y. et al., 2025). Secondly, it can slow down the inflammatory response of the nervous system and improve the function of glial cells (Liang et al., 2024). Furthermore, MSC-EVs promote the proliferation, migration, and angiogenic capabilities of brain microvascular endothelial cells, which helps restore neural function through angiogenesis following cerebral ischemic injury (Bao et al., 2024).

### 3.3 Respiratory diseases

The lungs are delicate organs that can be easily damaged by pathogens, harmful substances, and changes in the environment, which can result in various chronic diseases (Hu et al., 2024). The primary mechanisms of pulmonary diseases involve several processes: the infiltration of inflammatory cells, destruction of the alveolar structure, reduced clearance of alveolar fluid, release of cytokines and associated storms, airway remodeling, and the development of pulmonary fibrosis (Hoang et al., 2022). MSCs can inhibit alveolar cell apoptosis, promote alveolar cell proliferation, suppress inflammation, reduce oxidative stress, and inhibit fibroblast activity, which collectively helps to reduce pulmonary fibrosis (Feng et al., 2024). MSC-EVs have demonstrated therapeutic potential in a range of respiratory diseases, including acute lung injury (ALI), acute respiratory distress syndrome (ARDS), infectious diseases like coronavirus disease 2019 (COVID-19), chronic obstructive pulmonary disease, asthma, and idiopathic pulmonary fibrosis.

ALI occurs when the alveolar-capillary barrier is compromised or inflammatory responses are activated. This leads to alveolar cell dysfunction, protein edema in the alveolar space, and an accumulation of inflammatory cells (Hu et al., 2022). MSC-EVs contribute to the treatment of ALI by protecting alveolar cells, restoring the alveolar-capillary barrier, regulating immune responses, reducing inflammation, promoting fluid clearance, and eliminating microorganisms (Hu et al., 2022; Sun et al., 2022). For example, the downregulation of miR-181 induced by lncRNA-p21 may inhibit epithelial cell apoptosis and reduce lung tissue injury by increasing SIRT1 expression (Sui et al., 2021). Currently, research on the anti-inflammatory effects primarily concentrates on alveolar macrophages, building on the established roles of MSC-EVs in ALI treatment. For instance, activating miR-150-5p in the MAPK pathway promotes M2 polarization of macrophages and inhibits pro-inflammatory cytokines, which helps alleviate ALI (Zhao C. et al., 2024).

Asthma is characterized by increased mucus secretion in the airways, blood vessel dilation, fluid leakage from blood vessels, and the infiltration of various inflammatory cells. Other features include airway smooth muscle hyperplasia, reduced integrity of epithelial and cartilage structures, and excessive collagen deposition beneath the epithelium (Hu et al., 2024). MSC-EVs have immunoregulatory effects, including promoting the proliferation of regulatory T cells, reducing eosinophils, facilitating the transition of macrophage phenotype from pro-inflammatory M1 state to anti-inflammatory M2 state, and lowering Th2 cytokine levels; they can also inhibit lung inflammation, alleviate airway hyperreactivity (AHR), suppress bronchial smooth muscle proliferation, and inhibit airway remodeling, improving ventilatory dysfunction (Chen et al., 2024). For example, in a mouse model of asthma, paraoxonase-1 significantly reduced AHR, total inflammatory cell counts, and eosinophil levels in bronchoalveolar lavage fluid. It also diminished airway eosinophilic inflammation, reduced serum total IgE levels, and lowered Th2-mediated inflammation (Jung et al., 2024).

COVID-19 caused by severe acute respiratory syndrome coronavirus 2 (SARS-CoV-2) has caused severe damage worldwide (Rahmani et al., 2024). MSC-EVs operate through several mechanisms, such as: preventing cell death and oxidative damage, restoring damaged lung cells, reducing neutrophil activation, enhancing macrophage function, promoting monocyte M2 polarization, regulating T cell activity, managing inflammation, and preventing pulmonary fibrosis (Monguió-Tortajada et al., 2021). For example, extracellular vesicles from adipose-derived mesenchymal stem cells can reduce the replication of SARS-CoV-2 virus in cells, decrease the accumulation of inflammatory cells in the lungs of infected mice, and alleviate the thickening of alveolar septa, thereby reducing symptoms of lung injury (Lee et al., 2024). Additionally, Wharton Jelly-derived MSC vesicles (WJMSC-EVs) transferred miR-146a to recipient lung epithelial cells, rescuing epithelial-endothelial intercellular communication and decreasing the increase in immune cell recruitment through NF-κB pathway downregulation and inflammatory cytokine secretion (Anggraeni et al., 2024).

### 3.4 Cardiovascular diseases

Cardiovascular diseases are the leading cause of death globally, responsible for 31% of all deaths. As the population ages, cardiovascular diseases are becoming increasingly common, leading to a significant economic burden (Evans et al., 2020). Cardiovascular diseases are marked by myocardial damage, limited regenerative capacity, and progressive characteristics. While drug and surgical interventions can improve vascular health, they do not promote regeneration or functional recovery of peripheral tissues affected by cardiovascular diseases (Yun and Lee, 2019). MSC-EVs mainly manifest as inhibiting cardiomyocyte apoptosis and regulating immune responses to reduce inflammation, promote angiogenesis, enhance cardiac regeneration and repair capacity, and alleviate cardiac fibrosis, and are considered a very promising therapeutic strategy for cardiovascular diseases (Matta et al., 2022; Kumar et al., 2024). Currently, MSC-EVs show therapeutic value in various cardiovascular diseases, such as atherosclerosis, myocardial infarction, heart failure ischemia/reperfusion, aneurysm, pulmonary arterial hypertension, and diabetic retinopathy.

In myocardial infarction, MSC-EVs contribute to angiogenesis, reduce cell apoptosis, regulate immune responses, inhibit harmful ventricular remodeling, and enhance cardiac function (Tan et al., 2020; Liu Y. et al., 2023). Liu et al. found that hypoxic preconditioning of adipose-derived mesenchymal stem cells improves cardiac injury after MI by activating the circ-Stt3b/miR-15a-5p/GPX4 signaling pathway, reducing reactive oxygen species (ROS) levels and inflammatory factor expression, and decreasing ferroptosis (Liu et al., 2024). Additionally, another study found that miR-125b regulates several targets and prevents cardiomyocyte death. It also restricts fibroblast growth and counteracts myocardial remodeling after acute myocardial infarction, thereby promoting cardiac function recovery (Wang Z. et al., 2025).

Atherosclerosis is characterized by plaque formation, which consists of different cells, lipids, and debris within the vascular intima (Basatemur et al., 2019). MSC-EVs exert anti-inflammatory effects by polarizing macrophages, reducing their infiltration, and inhibiting eosinophil progression. They also alleviate damage from endothelial cell senescence and inhibit the activation, proliferation, and migration of vascular smooth muscle cells (Li T. et al., 2022; Olejarz et al., 2023; Comariţa et al., 2022). For instance, a study on atherosclerosis mice revealed that miR-674-5p inhibits H_2_O_2_-induced senescence and oxidative stress in primary mouse aortic endothelial cells by enhancing CTRP9 expression, which promotes their proliferation, migration, and angiogenesis (Zeng et al., 2024). In a mouse model of diabetes-related atherosclerosis, BMSC-EVs regulate macrophage polarization and autophagy via the AMPK/mTOR signaling pathway, which in turn inhibits the proliferation, migration, and foam cell formation of vascular macrophages (Liu et al., 2025).

### 3.5 Gastrointestinal diseases

Gastrointestinal diseases encompass various conditions that affect the gastrointestinal tract, which extends from the esophagus to the rectum. These diseases also involve accessory digestive organs, such as the liver, gallbladder, and pancreas. MSC-EVs demonstrate several beneficial effects, including regeneration, antioxidant activity, anti-inflammatory properties, prevention of apoptosis, and antifibrotic effects in various experimental models of gastrointestinal diseases (Didamoony et al., 2023). The current applications of MSC-EVs in treating gastrointestinal diseases primarily include conditions such as acute liver injury, liver fibrosis, liver failure, non-alcoholic fatty liver disease, acute pancreatitis, and inflammatory bowel disease (IBD).

Liver fibrosis is a long-lasting inflammatory response triggered by cytokines and chemokines, which leads to injury and apoptosis of hepatocytes. When hepatic stellate cells (HSCs) and macrophages are activated, they cause ECM protein accumulation, which accelerates fibrosis progression (Zhu et al., 2024; Wu et al., 2022). MSC-EVs can regulate several molecular pathways in target cells by reducing inflammation, liver macrophage activation, HSCs activation, fibrosis, hepatocyte apoptosis, and epithelial-mesenchymal transition (EMT) (Chiabotto et al., 2020). For instance, research indicates that delivering USP9X via MSC-EVs inhibits angiogenesis, mediated by the marker Angiogenin 2, and reduces liver fibrosis associated with metabolic dysfunction-related fatty liver hepatitis (Wang et al., 2024b). Additionally, WJMSC-EVs activated macrophages suppress immune responses and protect against liver fibrosis by directly inhibiting HSCs activation (Torabi et al., 2024).

IBD is a chronic inflammatory disease that can be divided into ulcerative colitis and Crohn’s disease according to etiology and pathogenesis (Cosnes et al., 2011). The pathogenesis of IBD involves genetics, the intestinal mucosal barrier, environmental factors, gut microbiota, and the immune system, among others (Wei et al., 2024). MSC-EVs primarily alleviate intestinal mucosal inflammation by inhibiting pro-inflammatory cytokines, increasing anti-inflammatory mediators, restoring the mucosal barrier, and preventing intestinal narrowing through fibrosis inhibition (Wang et al., 2022; Shen et al., 2022). For instance, hypoxic preconditioning of hair follicle mesenchymal stem cell exosomes helps alleviate mitochondrial dysfunction and boosts mitochondrial autophagy, thereby relieving ulcerative colitis by inhibiting the PI3K/AKT/mTOR signaling pathway (Li N. et al., 2024). Furthermore, in a rat model of perianal fistula associated with Crohn’s disease, injection of exosomes delivered by nanofiber-hydrogel composites effectively reduced inflammation at the fistula site and facilitated healing through tissue regeneration via macrophage polarization and neo-vascularization (Li L. et al., 2024).

### 3.6 Kidney diseases

Kidney diseases are often silent and complicated, making treatment difficult, and most types have no cure (Tang et al., 2022). MSC-EVs have therapeutic effects on several conditions, including acute kidney injury (AKI), chronic kidney disease (CKD), renal fibrosis, ischemia-reperfusion injury, diabetic nephropathy, and atherosclerotic renal vascular disease. Kidney injury is classified into two types based on the onset process: AKI and CKD. The pathophysiology of AKI involves tubular necrosis, interstitial edema, inflammation, and vascular changes. In contrast, CKD is characterized by glomerulosclerosis, tubular atrophy, interstitial fibrosis, renal ischemia, and capillary loss (Li B. et al., 2024).

MSC-EVs help prevent AKI by regulating angiogenesis, promoting M2 macrophage polarization, and decreasing macrophage infiltration. They do this by lowering apoptosis and oxidative stress levels while stimulating the proliferation and autophagy of renal tubular cells (Kosanović et al., 2022). For instance, miR-125b-5p inhibits p53 protein expression in renal tubular epithelial cells (TECs). This action rescues TECs from G2/M cell cycle arrest and apoptosis, improves ischemic AKI, and promotes tubular repair (Cao et al., 2021). Additionally, in a mouse model of sepsis-associated acute kidney injury (SAKI), HUCMSC-Exos deliver miR-375 to CD4^+^ T cells, thereby alleviating AKI in SAKI mice by promoting autophagy and inhibiting T cell apoptosis (Liu and Chen, 2024).

In the treatment of CDK, the mechanism of action of MSC-EVs not only regulates cell apoptosis, oxidative stress, and alleviates inflammation, but also exerts effects by improving microvascular injury and fibrosis (Eirin and Lerman, 2021). For example, research has found that BMSC-Exos inhibit cell apoptosis and calcification by targeting NFAT5 through miR-381-3p, thereby alleviating vascular calcification in mice with CKD (Liu et al., 2022). Additionally, in a mouse model of SAKI, HUCMSC-Exos deliver miR-375 to CD4^+^ T cells, thereby alleviating AKI in SAKI mice by promoting autophagy and inhibiting T cell apoptosis (Jin et al., 2021).

### 3.7 Skin diseases

The skin, the largest organ of the human body, acts as a physical barrier against the external environment, safeguarding internal organs, bones, muscles, and soft tissues from pathogenic microorganisms, chemicals, moisture loss, electrolyte imbalance, and temperature fluctuations (Tienda-Vázquez et al., 2023). MSC-EVs possess anti-inflammatory and immune-regulating effects, anti-aging properties, and they promote wound healing, stimulate hair growth, and repair the skin barrier (Ha et al., 2020). Current applications of MSC-EVs in skin diseases include wound healing (such as burns, trauma, and diabetic wounds), atopic dermatitis, psoriasis, alopecia, and skin aging (Quiñones-Vico et al., 2021).

Wound healing is a complex biological process that involves various tissues and cells working together to repair damaged structures. It occurs in four stages: hemostasis, inflammation, proliferation, and remodeling (Li Y. et al., 2024). MSC-EVs aid in repairing damaged structures by promoting the regeneration of the epidermis, dermis, hair follicles, nerves, and blood vessels. They also encourage macrophage polarization, wound angiogenesis, cell proliferation, and cell migration, while inhibiting excessive extracellular matrix production and scar formation through angiogenesis-related and anti-fibrosis pathways (Ding et al., 2023). For example, adipose-derived mesenchymal stem cell exosomes regulate macrophage polarization and enhance IL-33 release, driving keratinocyte proliferation, collagen deposition, and epithelialization through the Wnt/β-catenin signaling pathway, thereby promoting wound healing (Wang Y. et al., 2025). In the context of diabetic foot ulcers (DFU), circMYO9B promotes angiogenesis by regulating the hnRNPU/CBL/KDM1A/VEGFA axis, which accelerates the healing of DFU wounds (Wang Z. et al., 2024).

**FIGURE 3:**
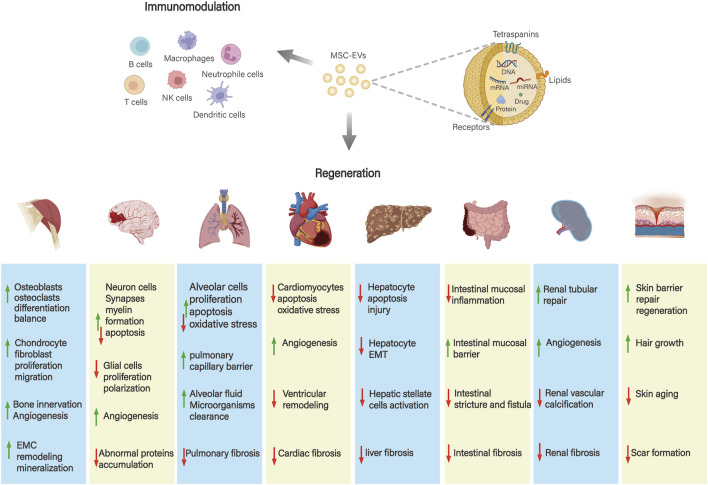
Application and mechanism of action of MSC-EVs in different systemic diseases. MSC-EVs play a role in musculoskeletal, neurological, respiratory, cardiovascular, gastrointestinal, hepatic, renal, and skin diseases mainly through immunomodulatory effects and promotion of tissue repair and regeneration.

The application and mechanism of action of MSC-EVs in the above diseases are demonstrated in Figure 3. MSC-EVs can also be applied to other regenerative diseases such as periodontal regeneration, retinal and corneal regeneration, infertility, premature ovarian failure, and preeclampsia during pregnancy.

## 4 Conclusion

So far, early results from registered clinical trials have confirmed the effectiveness of MSC-EVs in treating various conditions, such as COVID-19, ARDS, chronic airway inflammation, osteoarthritis, spinal cord injuries, stroke, anal fistulas, hair loss, and skin aging. Several trials have shown that using MSC-EVs can lower mortality rates associated with COVID-19 (Zamanian et al., 2024; Lightner et al., 2023). For example, a phase II multicenter double-blind randomized placebo-controlled trial at five U.S. sites included 102 patients with moderate to severe ARDS due to COVID-19. The study found that intravenous ExoFlo is safe and effectively reduces the 60-day mortality rate among participants (Lightner et al., 2023). Moreover, MSC-EVs enhance motor function and magnetic resonance imaging (MRI) indicators in patients with ischemic stroke (Bang et al., 2022). They are also safe and effective for treating complex perianal fistulas (Pak et al., 2023). Additionally, MSC-EVs can improve skin aging by reducing wrinkles and increasing elasticity, hydration, and pigmentation and treat mild to moderate hair loss (Park et al., 2023; Gentile et al., 2025). However, there are also clinical trials with poor results. For example, in the treatment of knee osteoarthritis, no improvement in clinical symptoms or MRI findings was observed compared to the placebo (Bolandnazar et al., 2024). This highlights the urgent need for more extensive, multicenter clinical trials to confirm the therapeutic effects of MSC-EVs in various treatment applications.

The clinical application of MSC-EVs encounters several challenges. These include methods for large-scale production and isolation, the pharmacokinetics, targeting mechanisms, and transport dynamics that direct EVs to their intended destinations. Additionally, safety profiles are crucial for determining optimal therapeutic dosages and assessing potential toxicity with repeated treatments. MSCs have a limited ability to grow indefinitely. As culture time and passage number increase, their proliferation rates decrease, and their morphology changes. This limitation hinders the large-scale production of EVs (Costela-Ruiz et al., 2022; Phinney and Pittenger, 2017). Currently, MSCs can be genetically modified to carry the MYC gene through transfection techniques. This process produces immortalized MSCs that proliferate more rapidly and exhibit reduced adhesion properties, thereby enhancing their production capabilities (Chen et al., 2011). Furthermore, using 3-dimensional (3D) printed scaffold perfusion bioreactor systems significantly enhances EVs secretion compared to traditional single-cell cultures of MSCs. This improvement leads to increased overall production efficiency (Holkar et al., 2022). For example, in a vertical wheel bioreactor, cultivating primary MSCs in a 3D environment using microcarrier suspension culture releases EVs. This method increases the EVs yield by 100-fold compared to traditional 2-dimensional (2D) cultures and shows higher biological activity (Otahal et al., 2024).

The short half-life of EVs, which are quickly eliminated from the body, presents a challenge for achieving lasting therapeutic effects (Leung et al., 2022). To overcome this challenge, hydrogels made from natural polymers, such as collagen and gelatin, as well as synthetic polymers like polyethylene glycol, are used to encapsulate drugs, particles, or cells. This approach enables controlled drug degradation, protects these substances from the *in vivo* environment, and allows for targeted therapeutic applications (Ju et al., 2023; Huang et al., 2022). Additionally, targeted therapy can be improved by incorporating specially designed peptides and nucleic acids into EVs and utilizing external magnetic fields (Kolenc and Maličev, 2024; Rezaie et al., 2022). For instance, magnetic nanoparticles containing iron oxide, derived from MSCs, have been shown to significantly improve targeting and therapeutic efficacy in ischemic lesions (Zhao S. et al., 2024).

The safety of MSC-EVs applications is a prerequisite for production and application. The International Society for Extracellular Vesicles (ISEV) position paper raises requirements for the application of extracellular vesicle therapy in clinical trials: donors and subjects should meet ethical requirements; the purity and impurities of the separation, as well as whether reagents and materials meet quality standards, should be considered; testing for microbial contamination, allergens, and other drug contaminants is necessary; considerations should include dosage, administration route, pharmacokinetics, and potential side effects such as toxicity (Lener et al., 2015).

This review highlights the unique advantages of MSC-EVs as natural bioactive carriers by comparing them with traditional stem cells. In addition, it elaborates in detail on their molecular mechanisms of action in different systemic disease models based on the latest literature reports. Furthermore, the review summarizes the current status of clinical trial applications and emphasizes the urgent need to improve standardized preparation methods, targeted delivery strategies, and precise dosing regimens. MSC-EVs are expected to revolutionize treatment models. They will provide a safe, multifunctional, and precisely targeted therapy. Ultimately, it will promote the paradigm shift of medical model from “disease treatment” to “system regulation” and open a new era of precision medicine.

## Glossary

ACL: anterior cruciate ligament
AD: Alzheimer’s disease
AHR: alleviate airway hyperreactivity
AKI: acute kidney injury
ALI: acute lung injury
ARDS: acute respiratory distress syndrome
Aβ: amyloid-β
BMSC-EVs: bone marrow mesenchymal stem cell-derived extracellular vesicles
BMSC-Exos: bone marrow mesenchymal stromal cell-derived exosomes
CKD: chronic kidney disease
COVID-19: coronavirus disease 2019
DAMPs: damage associated molecular patterns
ECM: extracellular matrix
EMT: epithelial-mesenchymal transition
EVs: extracellular vesicles
FTO: alpha-ketoglutarate dependent dioxygenase
HSCs: hepatic stellate cells
HUCMSC-Exos: human umbilical cord mesenchymal stem cells
IBD: inflammatory bowel disease
ISEV: International Society for Extracellular Vesicles
MRI: magnetic resonance imaging
MSCs: mesenchymal stem cells/mesenchymal stromal cells
MSC-EVs: mesenchymal stem cell-derived extracellular vesicles
MSDs: musculoskeletal disorders
PHLDA2: recombinant pleckstrin homology Like Domain Family A, Member two
ROS: reactive oxygen species
SAKI: sepsis-associated acute kidney injury
SARS-CoV-2: severe acute respiratory syndrome coronavirus two
TBI: traumatic brain injury
TECs: tubular epithelial cells
WJMSC-EVs: Wharton Jelly-derived mesenchymal stem cell-derived extracellular vesicles
2D: 2-dimensional
3D: 3-dimensional
